# Combined atomic force microscopy and photoluminescence imaging to select single InAs/GaAs quantum dots for quantum photonic devices

**DOI:** 10.1038/s41598-017-06566-5

**Published:** 2017-07-24

**Authors:** Luca Sapienza, Jin Liu, Jin Dong Song, Stefan Fält, Werner Wegscheider, Antonio Badolato, Kartik Srinivasan

**Affiliations:** 1 Department of Physics and Astronomy, University of Southampton, Southampton, SO17 1BJ UK; 2 Center for Nanoscale Science and Technology, National Institute of Standards and Technology, Gaithersburg, MD 20899 USA; 3 Maryland NanoCenter, University of Maryland, College Park, MD 20742 USA; 4 Center for Opto-Electronic Materials and Devices Research, Korea Institute of Science and Technology, Seoul, 136-791 South Korea; 5 Solid State Physics Laboratory, Swiss Federal Institute of Technology, 8093 Zurich, Switzerland; 6 Department of Physics and Max Planck Centre for Extreme and Quantum Photonics, University of Ottawa, Ottawa, Ontario K1N 6N5 Canada

## Abstract

We report on a combined photoluminescence imaging and atomic force microscopy study of single, isolated self-assembled InAs quantum dots. The motivation of this work is to determine an approach that allows to assess single quantum dots as candidates for quantum nanophotonic devices. By combining optical and scanning probe characterization techniques, we find that single quantum dots often appear in the vicinity of comparatively large topographic features. Despite this, the quantum dots generally do not exhibit significant differences in their non-resonantly pumped emission spectra in comparison to quantum dots appearing in defect-free regions, and this behavior is observed across multiple wafers produced in different growth chambers. Such large surface features are nevertheless a detriment to applications in which single quantum dots are embedded within nanofabricated photonic devices: they are likely to cause large spectral shifts in the wavelength of cavity modes designed to resonantly enhance the quantum dot emission, thereby resulting in a nominally perfectly-fabricated single quantum dot device failing to behave in accordance with design. We anticipate that the approach of screening quantum dots not only based on their optical properties, but also their surrounding surface topographies, will be necessary to improve the yield of single quantum dot nanophotonic devices.

## Introduction

Single self-assembled InAs/GaAs quantum dots (QDs) grown by molecular beam epitaxy (MBE)^1, 2^ are one of the most promising solid-state emitters for quantum technologies, due to their potential high stability and emission efficiency, easy on-chip integration, and coherence of the single-photon emission^3^. To preserve such characteristics, QDs have to be capped by larger bandgap semiconductors (e.g., GaAs), with layer thicknesses typically exceeding 50 nm^4^. To study the relation between material structure and optical properties of QDs, several techniques have been employed, including scanning probe microscopy^5–8^ of uncapped or partially capped QDs, and transmission electron microscopy of capped QDs^9, 10^. Advances in crystal growth have reached high structural control and material purity, allowing QDs to be successfully employed as gain media in lasers^11^ and as single artificial atoms in cavity quantum electrodynamics^3, 12^.

However, photonic devices (such as microcavities) that require a single emitter to be in a certain position and to emit at a specific wavelength are still very challenging to implement with high yield with these QDs, because the Stranski-Krastanov nucleation process at the origin of QD growth produces a random spatial positioning of the QDs across the wafer and inhomogeneous spectral broadening of the QD ensemble exciton emission. To achieve accurate positioning of single QDs within nanophotonic devices, two classes of techniques have been developed, one based on changes to the surface morphology in correspondence to the buried emitters, and the other based on their light emission. The former includes atomic force microscope (AFM) mapping^13^ and scanning electron microscopy^14, 15^ to detect surface deformations due to strain propagation from the buried QD, while the latter includes scanning confocal photoluminescence microscopy^16–18^, scanning cathodoluminescence^19^, and photoluminescence imaging^20, 21^. In this work, we combine techniques from each of these two classes in order to better understand the extent to which the optical performance of single QD nanophotonic devices might be influenced by the morphology of the crystal structure surrounding the QD.

**Figure 1:**
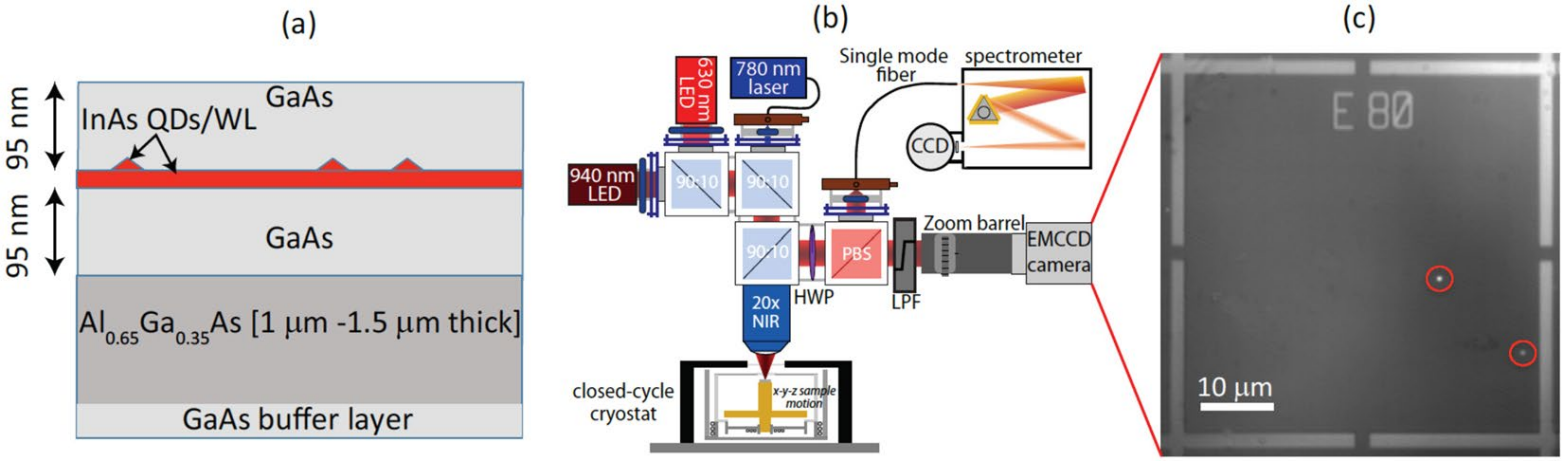
Sample structure and experimental set up (**a**) Schematic of the sample under study (not to scale), comprising a single layer of InAs quantum dots (red triangles), grown on an InAs wetting layer (WL) between two 95 nm thick layers of GaAs, and situated on top of a 1 *μ*m or 1.5 *μ*m thick Al_0.65_Ga_0.35_As layer on a GaAs buffer layer followed by a GaAs substrate. (**b**) Schematic of the photoluminescence setup. An infrared light emitting diode (LED, emission centered at 940 nm) is used for illumination of the sample while either a 630 nm red LED or a 780 nm laser is used for excitation of the quantum dots (QDs), depending on whether excitation over a broad area (LED) or of individual QDs (laser) is required. Samples are placed within a cryostat on an x-y-z positioner. Imaging is done by directing the emitted and reflected light into an Electron Multiplied CCD (EMCCD) camera, while spectroscopy is performed by collecting emission into a single-mode fiber and sending it to a grating spectrometer. (**c**) EMCCD image of the photoluminescence from two QDs (highlighted by red circles) and reflected light by the alignment marks (metallic crosses), acquired by illuminating the sample simultaneously with both the red and near-infrared LEDs, at a temperature of 4 K.

The presence of surface features in QD epitaxy is not surprising, given the number of interfaces present in a layer structure such as that shown in Fig. 1(a), and the potential for defects to form at interfaces and influence (through strain) the growth of subsequent layers. Here, the InAs QD layer is sandwiched between two GaAs layers, which are grown on a thick Al_0.65_Ga_0.35_As layer (used as a sacrificial layer in device fabrication) that is grown on a GaAs substrate. The coherent deformation of the crystal caused by the InAs/GaAs lattice mismatch produced when growing the QD layer is intrinsic and appears on the surface as a shallow island with sub-micrometer diameter. In contrast, defects formed well below the InAs layer, close to the GaAs substrate/epilayer interface, are usually buried by thick lattice-matched buffer layers (e.g., the Al_0.65_Ga_0.35_As layer) and can recover the crystal coherence, appearing at the surface as large convex oval defects^22^. Because nucleation of InAs is energetically favorable close to crystal steps, QDs tend to decorate the edge of the oval defects. The coherent deformations of the crystal surrounding a QD and propagating up to the surface can strongly affect the QD emission properties without necessarily affecting the optical quality (e.g, as judged by the emission linewidth or number of emitting states). This aspect is often neglected in experiments relying on random choice of the target QD, but may be important when considering the behavior of the QD within a surrounding photonic structure. In some cases, such crystal deformation can have positive benefit. For example, in ref. 23, the authors attributed an increased emission intensity to the presence of an unclassified oval defect close to the QD, acting in a similar way as a solid immersion lens and thus increasing the extraction efficiency of the single photons emitted by the QD^24^. On the other hand, crystal defects may degrade the performance of nanofabricated photonic structures, such as photonic crystals, which are used to enhance radiative rates and extraction efficiency, but whose characteristic lengths are at the sub-micrometer scale, and are thus comparable to the surface defect sizes.

Here, we present a study of the surface morphology of GaAs samples containing single QDs and investigate the correlation between surface features and emitter locations. This is made possible by the implementation of a photoluminescence imaging technique that we have recently developed, based on a double light-emitting diode (LED) illumination scheme (see Fig. 1(b)). By using a red LED to excite the QD emission and near-infrared LED to illuminate alignment marks deposited on the sample’s surface, we are able to locate single QDs with uncertainties below 30 nm^20^. Such a technique allows us to optically characterize the emitters and find their positions (see Fig. 1(c)), and then to investigate the nearby surface morphology of the sample by means of AFM. By combining photoluminescence and AFM techniques, we are able to correlate the position of the QDs with respect to the surface features observed. While the present work does not focus on the materials science motivation for the formation of surface defects and the quantum dot position with respect to those, it does provide a technique that can be applied to get insights, at the single quantum dot level, into quantum dot nucleation processes under different growth conditions, and their influence on quantum dot optical properties.

Using our combined optical positioning and AFM scanning approach, we find that a significant fraction (between 20% and 60%, depending on the wafer) of isolated quantum dots we study are in proximity to large topographic features. However, we find the emission spectra of the quantum dots in our wafers are qualitatively similar regardless of whether they are in proximity of such large (*μ*m-scale) surface defects. Because such surface defects can strongly modify the properties of the photonic devices (e.g., in terms of modal characteristics such as resonant wavelength), it therefore appears to be important to characterize both the QD’s optical properties and the surrounding surface morphology when screening candidate QDs for subsequent incorporation in devices. In particular, typical non-resonant photoluminescence spectroscopy seems to be, by itself, an inadequate approach for identifying candidate QDs for nanophotonic devices, such as photonic crystals and grating cavities.

## Results

We have investigated four QD samples (labeled as samples I–IV) presenting low QD densities (about 1 to 10 QDs per 1000 *μ*m^2^), all with the same nominal structure (see Fig. 1(a)) and emitting at wavelengths between 900 nm and 1000 nm, but grown in different MBE chambers. We focus on such ultra-low QD density materials due to their specific relevance to quantum photonic experiments in which, for example, the interaction between only one QD and a confined optical mode must be ensured. Previous experiments operating in this regime include studies of strong coupling cavity QED^25^ and triggered single-photon generation^20^.

Samples I, III, and IV were grown by MBE at ETHZ. We used cracked As tetramer and a GaAs growth rate of 2.2 Å/s. The Ga cell featured a dual filament and was operated with hot tip. The substrate temperature during the growth of the 160 nm GaAs buffer layer was kept above 600 °C and it was lowered to 580 °C during the growth of the 190 nm GaAs device layer. The 1 *μ*m thick AlGaAs sacrificial layer was either grown by digital alloy (samples I and III) or by co-growth, changing the Ga cell temperature (sample IV). The AlGaAs growth rate was always below 2.8 Å/s. The QDs were grown at 520 °C and the rotation of the substrate was stopped to create a gradient in the QD density over the wafer. Sample II was grown by MBE at KIST. We used cracked As tetramer and a GaAs growth rate of 1.4 Å/s (Ga cell 1). The two Ga cells featured a dual filament and were operated with hot tip. The substrate temperature during the growth of the GaAs layers (200 nm thick buffer layer and 190 nm thick device layer) and 1.5 *μ*m thick AlGaAs layer was kept at 590 °C. The growth rates for the AlGaAs sacrificial layer, created through co-growth, were 0.68 Å/s for GaAs (Ga cell 2) and 1.47 Å/s for AlAs. The QDs were grown at the substrate temperature of 510 °C and the rotation of the substrate was stopped to create a gradient in the QD density over the wafer.

**Figure 2:**
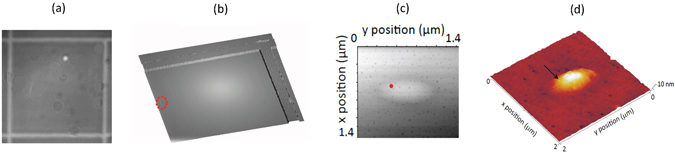
Example of combined photoluminescence and atomic force microscopy to correlate quantum dot location and surface morphological features (**a**) EMCCD image of the photoluminescence from a single QD and reflected light by the alignment marks (metallic crosses), acquired by illuminating the sample simultaneously with both the red and near-infrared LEDs, at a temperature of 4 K. (**b**) Atomic force microscope image of the area between two alignment marks (top right corner of panel (a)). (**c**,**d**) Atomic force microscope images of a 10 nm-high surface oval defect (found in correspondence to the dashed red circle in panel (b)) on which the position of the QD, measured from the photoluminescence image (red symbol in panel (c) and arrow in panel (d)), is shown. The one standard deviation uncertainty in the position of the QD is estimated to be 17 nm (see main text). [Sample I].

After locating the emission of single QDs using the photoluminescence imaging method (see Fig. 2(a)), we investigate the surface morphology in the nearby area via AFM (see Fig. 2(b)). The AFM images that we obtain show different surface features and an example is the oval defect shown in Fig. 2c and d (sample I). Such defects are attributed to GaAs droplets that can be formed during MBE growth^23, 26^ and are assumed to appear because of Ga cell spitting^27^. The presence of a variety of surface defects in MBE grown GaAs samples has been extensively reported (see, for instance, refs 28–31) and attributed mostly to the operation and geometry of the Ga cell. In our technique, we first acquire a photoluminescence image of the sample so as to determine the location of the QD emitting dipole with respect to the middle point of the two nearest alignment marks (Fig. 2(a)). Then, the sample’s surface, in correspondence to the area where the QD had been optically located, is mapped by using an AFM equipped with a tetrahedral, point-terminated, silicon cantilever with a tip radius of 7 nm, spring constant of 26 N/m and resonance frequency of 300 kHz (nominal values), in tapping mode (Fig. 2(b)). The sample is aligned in such a way that the scanning tip direction is orthogonal to one of the alignment marks. Two orthogonal scans are then collected (tip scanning at 0° and 90° angles) in order to be able to image the edges of the two orthogonal alignment marks with high accuracy. When a surface feature is observed, the position of the QD (distance from the center of the alignment marks) extracted from the optical images is marked on the AFM image (Fig. 2(c)). If needed, a zoomed-in scan is collected and the position of the surface feature with respect to the alignment marks is recovered by taking into account the scanning offset introduced in the process (Fig. 2(d)). We estimate the uncertainty in specifying the position of the emitting dipole in the AFM scan (Fig. 2(c)) based on the combined one standard deviation uncertainty of locating features in the optical positioning technique and the AFM scan. For the optical positioning technique, the location of the emitting dipole with respect to the center of the nearest alignment mark is determined from Gaussian fits to line cuts through the optical image^20^. For the AFM data, the alignment mark center is determined from Gaussian fits to the two peaks found in the first derivative of a line cut through the AFM image. For the data shown in Fig. 2(c), the imaging and AFM uncertainties are 11 nm and 13 nm, respectively, giving a combined uncertainty of 17 nm.

In ref. 23, the authors attribute the improved emission properties of the QD under study to its self-alignment to the center of a surface oval defect. By using our combined optical positioning-AFM approach, we are able to locate the QD emitting dipole with respect to the surface topography. For the oval defect shown in Fig. 2(c) and (d), we see that the emitter is located at the periphery of the oval defect. By carrying out photoluminescence measurements on the QD, under laser excitation, we do not observe a higher brightness compared to other emitters on the same sample, possibly due to the misalignment of the QD with respect to the center of the oval defect that we observe.

**Figure 3:**
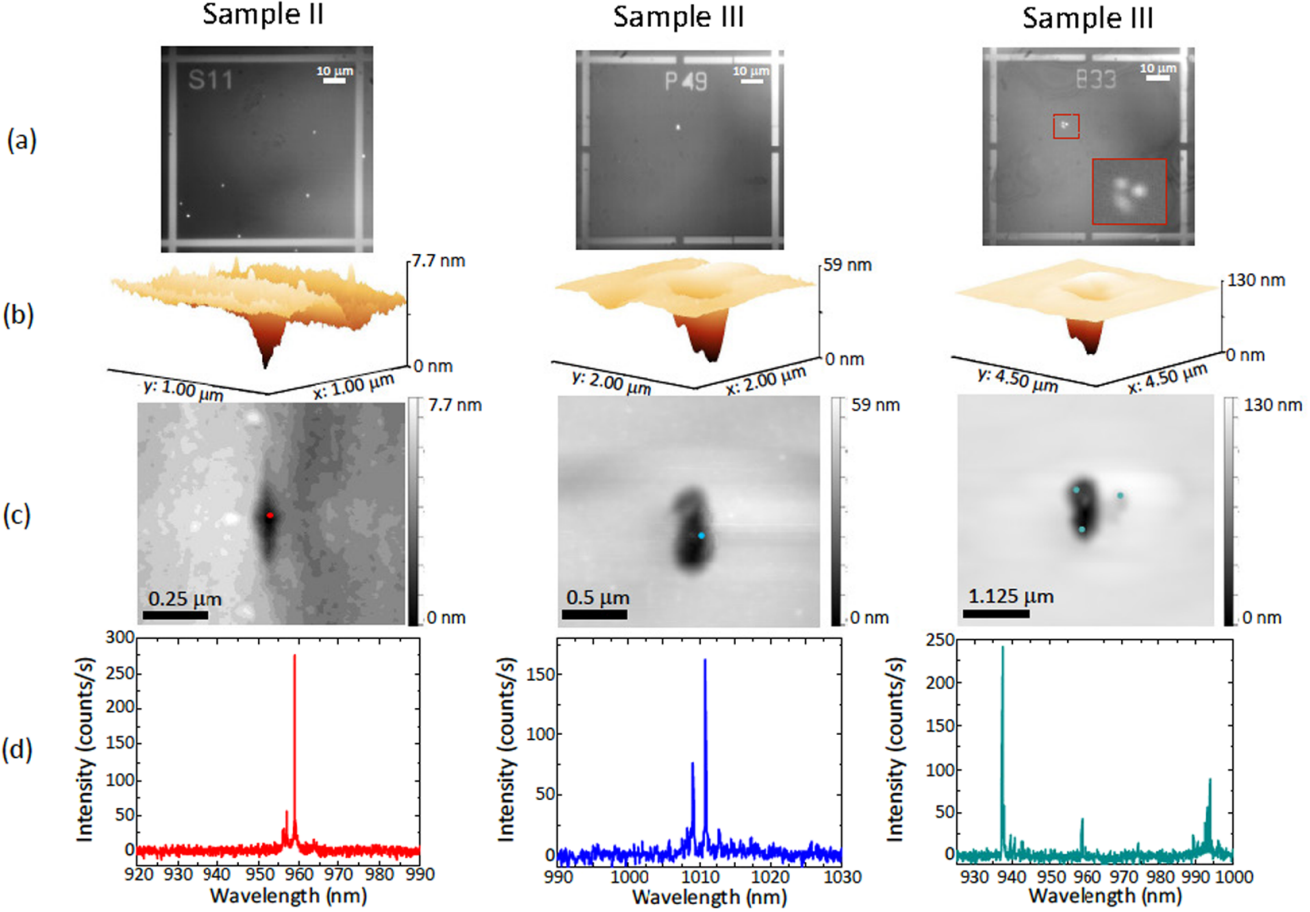
Examples of different morphological features observed via atomic force microscopy, correlated to quantum dot positions and their photoluminescence spectra (**a**) EMCCD images of the photoluminescence from QDs and reflected light by the alignment marks (metallic crosses), acquired by illuminating three different fields of two different samples (sample II in the first column and sample III in the second and third columns) simultaneously with both the red and near-infrared LEDs, at a temperature of 4 K. The inset in the third image represents a zoom-in of the area marked by the dashed red lines in the QD photoluminescence image. (**b**) Three-dimensional atomic force microscope images of surface features located in proximity to the QD’s emitting dipole positions, obtained from the photoluminescence images shown in panel (a) (the images are rotated 50° counterclockwise with respect to the images in panel (c)). (**c**) Two-dimensional atomic force microscope images of the surface features shown in panel (b). The colored dots represent the QD locations, as extracted from the images in panel (a). The one standard deviation uncertainties in the QD location are 47 nm (Sample II, left panel), 31 nm (Sample III, center panel), and 21 nm, 51 nm, and 48 nm (Sample III, right panel, top left, bottom left, and top right QDs, respectively). (**d**) Photoluminescence spectra collected from the QDs shown in panel (a), collected under 780 nm continuous-wave laser excitation at a temperature of 4 K, on a silicon CCD camera.

When measuring two other samples (labeled II and III), we see different surface features in correspondence to QD locations. It is worth noting that each kind of feature is peculiar to a specific wafer growth. Figure 3 shows examples of photoluminescence and AFM images, as well as the corresponding photoluminescence spectra collected from the specific QDs under study. The photoluminescence spectra are all characterized by sharp emission lines, as expected for high quality single QDs. In the first column (sample II), an example of a shallow and sharp dip in the AFM image is observed: by overlaying the position obtained from the photoluminescence image in panel (a) to the AFM image, we can see that the QD is aligned with the lowest portion of the dip. The second and third columns show surface features observed in a different sample (III), characterized by larger and more circular crater-like features. As shown in the third column, when larger AFM features are observed, several single QDs or clusters are likely to be seen. The presence of several optically active QDs is also confirmed by the photoluminescence spectra, where three groups of sharp peaks are visible (see Fig. 3(d)).

Similar topographical features are repeatedly observed for the same sample growth (oval defects in sample I, sharp shallow dips in sample II and larger crater-like features in sample III). In all cases, the sizes of the AFM features are too large to be caused solely by the presence of the buried QDs. Instead, it seems likely that the features are indicative of other defects that occur during the MBE growth process. This could include oval defect protrusions (sample I) that result from Ga droplets, for example, or various types of indentations that have been observed in GaAs MBE growth^28^. Such defects, if created prior to the InAs deposition step, might propagate upwards and seed QD nucleation, in a manner analogous to that which has been deterministically exploited in the growth of site-controlled QDs on patterned surfaces^32–34^. This could then explain the correlation between QD emission and topographic features in the AFM images. It is worth noting that the QD containing layer is grown on top of a thick AlGaAs layer (in place for subsequent undercut processes when fabricating devices), and that the growth of high Al-content AlGaAs layers can be one source of defects^35^.

In Table 1, the results of our study are summarized: we show the number of quantum dots measured, the kind of feature observed and the number of quantum dots found in correspondence of a surface feature. We classify as oval defects the morphological features similar to the one shown in Fig. 2d, presenting micron scale protrusions with respect to the surrounding material. An example of dip defect is shown in Fig. 3b and c (first column), characterised by a sharp fissure-like structure with sizes typically below 0.3 *μ*m by a few tens of nm. Crater-like features are shown in Fig. 3b and c (second and third column): these are about 1 *μ*m by a few hundred of nm in size, with more irregular shapes than dip defects. The observed features occur several times across multiple samples: a significant fraction of the examined locations, ranging from about 20% to 60%, shows surface topographical features that correspond with the QD locations, thus implying that such topographical defects are not solely responsible for QD nucleation but in many cases might behave as catalysts to the growth process.

**Table 1:** Summary table of the photoluminescence/atomic force microscopy results obtained from the measurements carried out on the different samples under study.

Sample	No. of QDs analyzed	AFM feature type	No. of matching AFM/QD positions
I	25	oval	7
II	21	dip	5
III	33	crater	19
IV	10	oval	6

The number of QDs that have been analyzed (photoluminescence positioning, spectral characterisation, and atomic force microscopy), the type of surface features observed with the atomic force microscope (examples of “oval, “dip” and “crater” features are shown in Figs 2d, 3c (first column), 3c (second column) respectively), and the number of instances in which the stated AFM surface feature type was observed in proximity to the QD’s emitting dipole are shown.

**Figure 4:**
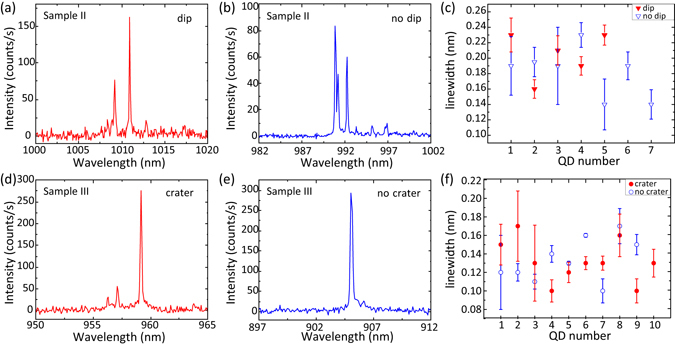
Comparison of the spectral linewidths measured from quantum dots found in correspondence to surface morphological features and smooth surfaces. (**a**) and (**d**): Photoluminescence spectra, collected under non-resonant laser excitation at a temperature of 4 K, from single QDs aligned with surface features [dip-like (Sample II) in (**a**) and crater-like (Sample III) in (**d**)]. (**b)** and (**e**): Photoluminescence spectra from single QDs, collected from the same samples as the corresponding spectra in (**a**) and (**d**), but from areas not showing any surface features. (**c**) and (**f**): Linewidths, extracted from Lorentzian fits of single emission lines measured in photoluminescence spectra like the ones shown in panels (a), (b), (d), and (e), of QDs found in correspondence to morphological features (filled red symbols) and to smooth surfaces (open blue symbols). The error bars are obtained from one standard deviation uncertainties in the Lorentzian fits.

As discussed in the next section, these surface features are large enough to potentially influence nanophotonic geometries that might be subsequently fabricated to exercise control on the QD emission pattern and lifetime (e.g., microresonators and photonic crystals). However, they have no apparent influence on the basic QD emission properties under non-resonant excitation. Photoluminescence spectra, collected from single emitters in areas where detectable surface features are both visible and absent, are shown in Fig. 4 for QDs from samples II and III. The linewidths that we measure do not show consistent differences for QDs found in correspondence to morphological features and those found in correspondence with smooth surfaces, as shown in Fig. 4c and f. For sample III, the average linewidth of QDs near craters is 0.13 nm ± 0.02 nm, and the same average linewidth of 0.13 nm ± 0.02 nm is recorded for QDs near smooth surfaces. For sample II, the overall average linewidth is larger, but still, there is no significant difference between QDs near dips (0.20 nm ± 0.03 nm) and near smooth surfaces (0.18 nm ± 0.03 nm).

Given that, from photoluminescence measurements, it is not possible to determine which emitter is aligned with potentially large and pronounced surface features, we anticipate that our approach, combining optical and AFM characterization, may be valuable in selecting single QDs to be embedded within optimized nanophotonic devices for quantum information technology applications.

## Discussion

The importance of the surface topography surrounding a QD when fabricating photonic devices for quantum information technology applications is multifold. Potentially, it might influence the coherence and quality of the quantum dot emission. While our current measurements indicate that, at the level of basic non-resonant spectroscopy, this is not the case, we note that other studies have shown that emission linewidth can be affected when the QD is close to surfaces^4, 36^. The presence of defects can also induce blinking^37^ and spectral diffusion^38^ in the QD emission. Even assuming that, as our measurements so far indicate, the QD is unaffected by such large surface topographical defects, their presence can dramatically influence the properties of the photonic device geometries often fabricated to enhance their properties. This is particularly relevant when dealing with nanoscale devices, such as photonic crystals or dielectric gratings, where the device features can be comparable to the length scale of the surface defects.

**Figure 5:**
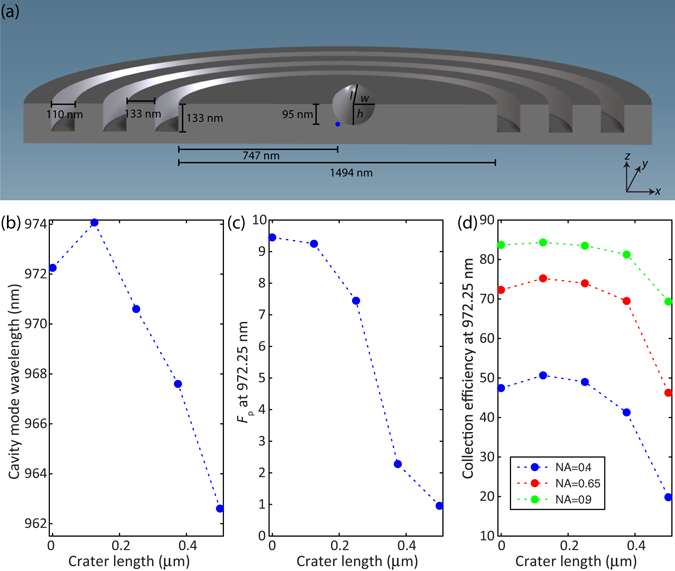
Finite-Difference Time-Domain simulations of the optical properties of a circular grating cavity containing a quantum dot and a crater-like feature close to its center. (**a**): Geometry of the simulated sample: the quantum dot (blue sphere) is positioned in the center of the circular grating structure. The center position of the half-ellipsoidal crater, used to depict a surface morphological (crater-like) feature, is fixed at a position ***x*** = 75 nm, *y* = 0 nm, *z* = 95 nm with respect to the QD. The crater width *w* is kept fixed at 125 nm, while the crater length *l* and crater height *h* have a fixed aspect ratio of 5:1, with the length *l* varying between 125 nm and 500 nm. The leftmost point (crater length = 0) corresponds to the circular grating structure in absence of a crater. Finite-difference time-domain simulations of the circular grating cavity wavelength (panel (b)), the Purcell factor *F*_*p*_ (panel (c)) and collection efficiency of the QD emission into objectives with different numerical aperture (NA) (panel (d)), plotted as a function of crater length.

To verify this, we carry out finite-difference time-domain simulations of one specific geometry, the circular grating ‘bullseye’ microcavity (e.g., from ref. 20), and include surface defects of an idealized shape (semi-ellipsoidal craters) and similar length scale as those observed in our experiments (see Fig. 5a for more specific details on the device geometry simulated). The results indicate that such a defect can induce a shift in the optical cavity resonant wavelength of up to 10 nm with respect to an unperturbed structure (see Fig. 5b). Such a large shift implies that a nominally perfectly-fabricated device would be far off-resonance with respect to the QD emission wavelength for which it was designed, which would completely change its performance (e.g., in terms of extraction efficiency, far-field distribution, and radiative lifetime) and reduce or completely suppress the Purcell factor, as shown in Fig. 5c. In Fig. 5d, we consider how these defects can influence the fraction of emitted photons that can be collected by various numerical aperture lenses (NA = 0.4, 0.65, and 0.9). We find that, while this value is relatively insensitive for the smaller-sized defects, a significant reduction is observed for larger defects, with feature sizes similar to the crater-like defects seen from Sample III (Fig. 3c). The reduction in efficiency is particularly pronounced for the smallest collection angle (NA = 0.4), an indication of how the defect can distort the far-field emission pattern and broaden its divergence angle. This would be of particular concern when coupling into single mode fibers, for example. Finally, we emphasize that the difference in total collected power will depend on both the Purcell factor and the collection efficiency. Thus, for NA = 0.4, a crater-like defect such as that seen in sample III can cause a drop in collected power by >20x.

Thus, our combined photoluminescence-AFM technique can be applied to select suitable QD emitters to be integrated into photonic devices, by pre-screening not just through optical measurements, but also through the surface topography. Such an approach is expected to be beneficial for increasing the yield of fabricated photonic devices with optimal performance since it allows one to map the sample’s surface quality and discard those QDs that appear in correspondence to morphological features that would negatively affect the optical performance of the fabricated device. In addition, we note that once a QD is located, more advanced AFM techniques, such as multifrequency AFM to study subsurface properties^39^ or contact mode force measurements^40^ to assess indentation levels and therefore strain properties, could also be implemented to extract structural information on the properties of buried QD samples.

### Data availability statement

The datasets generated during and/or analysed during the current study are available from the corresponding author on reasonable request.
